# Preoperative plasma growth-differentiation factor-15 for prediction of acute kidney injury in patients undergoing cardiac surgery

**DOI:** 10.1186/s13054-016-1482-3

**Published:** 2016-10-08

**Authors:** Matthias Heringlake, Efstratios I. Charitos, Kira Erber, Astrid Ellen Berggreen, Hermann Heinze, Hauke Paarmann

**Affiliations:** 1 Department of Anesthesiology and Intensive Care Medicine, University of Lübeck, Ratzeburger Allee 160, D - 23538 Luebeck, Germany; 2 Department of Cardiac Surgery, Martin-Luther University, Halle, Germany; 3 Department of Cardiac Anesthesiology, HELIOS – Clinic, Schwerin, Germany

**Keywords:** Cardiac surgery, Acute kidney injury, Biomarkers, Growth-differentiation factor-15, Cleveland Clinic Acute Renal Failure score, Euroscore

## Abstract

**Background:**

Growth-differentiation factor-15 (GDF-15) is an emerging humoral marker for risk stratification in cardiovascular disease. Cardiac-surgery-associated acute kidney injury (CSA-AKI), an important complication in patients undergoing cardiac surgery, is associated with poor prognosis. The present secondary analysis of an observational cohort study aimed to determine the role of GDF-15 in predicting CSA-AKI compared with the Cleveland-Clinic Acute Renal Failure (CC-ARF) score and a logistic regression model including variables associated with renal dysfunction.

**Methods:**

Preoperative plasma GDF-15 was determined in 1176 consecutive patients undergoing elective cardiac surgery. Patients with chronic kidney disease stage 5 were excluded. AKI was defined according to Kidney-Disease-Improving-Global-Outcomes (KDIGO) - creatinine criteria. The following variables were screened for association with development of postoperative AKI: age, gender, additive Euroscore, serum creatinine, duration of cardiopulmonary bypass, duration of surgery, type of surgery, total circulatory arrest, preoperative hemoglobin, preoperative oxygen-supplemented cerebral oxygen saturation, diabetes mellitus, hemofiltration during ECC, plasma GDF-15, high sensitivity troponin T (hsTNT), and N-terminal prohormone of B-type natriuretic peptide (NTproBNP).

**Results:**

There were 258 patients (21.9 %) with AKI (AKI stage 1 (AKI-1), n = 175 (14.9 %); AKI-2, n = 6 (0.5 %); AKI-3, n = 77 (6.5 %)). The incidence of AKI-1 and AKI-3 increased significantly from the lowest to the highest tertiles of GDF-15. In logistic regression, preoperative GDF-15, additive Euroscore, age, plasma creatinine, diabetes mellitus, and duration of cardiopulmonary bypass were independently associated with AKI. Inclusion of GDF-15 in a logistic regression model comprising these variables significantly increased the area under the curve (AUC 0.738 without and 0.750 with GDF-15 included) and the net reclassification ability to predict AKI. Comparably, in receiver operating characteristic analysis the predictive capacity of the CC-ARF score (AUC 0.628) was improved by adding GDF-15 (AUC 0.684) but this score also had lower predictability than the logistic regression model. In random forest analyses the predictive capacity of GDF-15 was especially pronounced in patients with normal plasma creatinine.

**Conclusion:**

This suggests that preoperative plasma GDF-15 independently predicts postoperative AKI in patients undergoing elective cardiac surgery and is particularly helpful for risk stratification in patients with normal creatinine.

**Trial registration:**

NCT01166360 on July 20, 2010.

**Electronic supplementary material:**

The online version of this article (doi:10.1186/s13054-016-1482-3) contains supplementary material, which is available to authorized users.

## Background

Cardiac-surgery-associated acute kidney injury (CSA-AKI) is an important and frequent complication in patients undergoing cardiac surgery and associated with increased morbidity and short-term and long-term mortality [1]. The incidence of CSA-AKI is variable and depends on the definition used, but has been reported to be as high as 40 % according to the Acute Kidney Injury Network (AKIN) criteria [2]. CSA-AKI requiring temporary renal replacement therapy occurs in up to 30 % of patients and has been associated with a mortality rate up to 60 % [1, 3].

No specific treatment for the prevention of CSA-AKI is available [4]. This may be related to the multifactorial pathophysiology of this complication [5], including postoperative factors that are difficult to predict preoperatively [6], but also to the fact that sparse modalities for preoperative risk stratification are available and that commonly used risk scores have variable prognostic utility in this regard [7]. However, preoperative identification of patients with a high risk of developing CSA-AKI is a prerequisite for developing strategies to ameliorate or prevent perioperative renal injury.

Very recently, two studies in 32 and 134 patients, respectively, provided evidence that the preoperative plasma level of the hormone growth-differentiation factor-15 (GDF-15) predicts postoperative renal injury [8, 9] in patients undergoing coronary artery bypass graft (CABG). We have previously shown that the preoperative plasma concentration of GDF-15 is an independent predictor of morbidity and short-term and long-term mortality in patients undergoing cardiac surgery [10]. The present study aims to confirm the findings of the pilot studies [8, 9] in a larger and heterogenous patient cohort to determine if this hormone may also be used for assessing the risk of developing AKI in this population.

## Methods

The present study is a secondary analysis of a large prospective observational cohort study analyzing the prognostic relevance of preoperative cerebral oxygen saturation and markers of cardiopulmonary dysfunction with respect to clinical outcomes in patients undergoing cardiac surgery [10, 11]. In total 2009 patients were screened during the study period between January and December 2008 and April to December 2009. There were 5 patients who refused to participate in the study, and 76 patients had their surgery cancelled. Complete datasets including GDF-15 measurements were available from 1458 patients and used for the previously published analyses [10]. Excluding emergency patients, off-pump revascularization, interventional procedures, and patients with chronic kidney disease stage 5, plasma samples for determination of plasma GDF-15 were available from 1176 consecutive patients undergoing elective cardiac surgery, and these were used for the present analysis.

The primary objective was to determine the relationship between preoperative plasma GDF-15 and AKI [12] in comparison with the Cleveland Clinic acute renal failure (CC-ARF) score [13] and a comprehensive logistic regression model based on variables typically associated with AKI in patients undergoing cardiac surgery, to investigate whether GDF levels can further improve risk stratification for AKI.

Plasma samples for determination of GDF-15 were taken immediately preoperatively (before induction of anesthesia) and determined as described recently [10]. Plasma was separated and stored at -80 °C for further analysis. Analyses were accomplished within 6 months after completion of enrollment by electrochemiluminescence immunoassays using Elecsys 2010 analyzers (Roche Diagnostics, Mannheim, Germany).

Plasma creatinine was measured the day before surgery. Postoperative AKI was graded according to the Kidney Disease Improving Global Outcomes (KDIGO) - creatinine criteria, [12] from maximal postoperative plasma creatinine in relation to the preoperative baseline, and from the need for renal replacement therapy (for grade 3 AKI). Cardiac surgery was performed with cardiopulmonary bypass (CPB) during moderate hypothermia. Surgical, anesthetic and CPB management have been described elsewhere [6, 10, 11]. Shortly, general anesthesia was induced with propofol and sufentanil, and before and after CPB was maintained with remifentanil and sevoflurane. During CPB, anesthesia was maintained with remifentanil and propofol. Perioperative fluid therapy was performed with balanced cristalloid solutions (Sterofundin ISO®, B.Braun, Melsungen, Germany) and 6 % hydroxyethyl starch 130/0.4 (Voluven®) (Fresenius Kabi, Bad Homburg, Germany). The CPB was primed with cristalloid.

## Statistical analyses

Analyses were performed with R version 3.2.2 (Development Core Team; 2015 R: a language and environment for statistical computing. R Foundation for Statistical Computing, Vienna, Austria. ISBN 3-900051-07-0, http://www.R-project.org/. Accessed 18 Sept 2016). Data are presented as mean ± SD if normally distributed or otherwise as median and 25 and 75 % quartiles. Comparisons between groups for univariate predictors of outcome were performed using the two-sided chi-square test for categorical variables and the Mann-Whitney or Kruskal-Wallis test for continuous variables, where appropriate.

The following variables were screened for association with the development of postoperative AKI: age, gender, additive Euroscore, serum creatinine, duration of CPB, duration of surgery, type of surgery, total circulatory arrest, preoperative hemoglobin level, preoperative oxygen supplemented cerebral oxygen saturation (ScO_2_), diabetes mellitus, hemofiltration during CPB, plasma GDF-15, high sensitivity troponin T (hsTNT), and N-terminal prohormone of B-type natriuretic peptide (NTproBNP).

The association between the aforementioned variables and the development of CSA-AKI was investigated using logistic regression and machine learning techniques. Model building and variable selection was performed using computer intensive methods (bootstrap aggregation) [14]. In order to investigate nonlinear effects and complex interactions among variables, machine learning methods were utilized (random forests and recursive partitioning using conditional inference trees) [15]. Variable importance (VIMP) and minimal tree depth was used to access the strength of association between each predictor and the development of AKI. Net reclassification improvement and integrated discrimination improvement to assess the additive predictive ability of GDF-15 on the development of AKI were calculated as described by Pencina et al. [16]. Comparisons between receiver-operating characteristic (ROC) curves were performed by the DeLong method and the bootstrap method. The *p* values (two-tailed) for the DeLong method are presented. Statistical significance was assessed at the 5 % level (*p* < 0.05 was considered statistically significant).

## Results

**Fig. 1:**
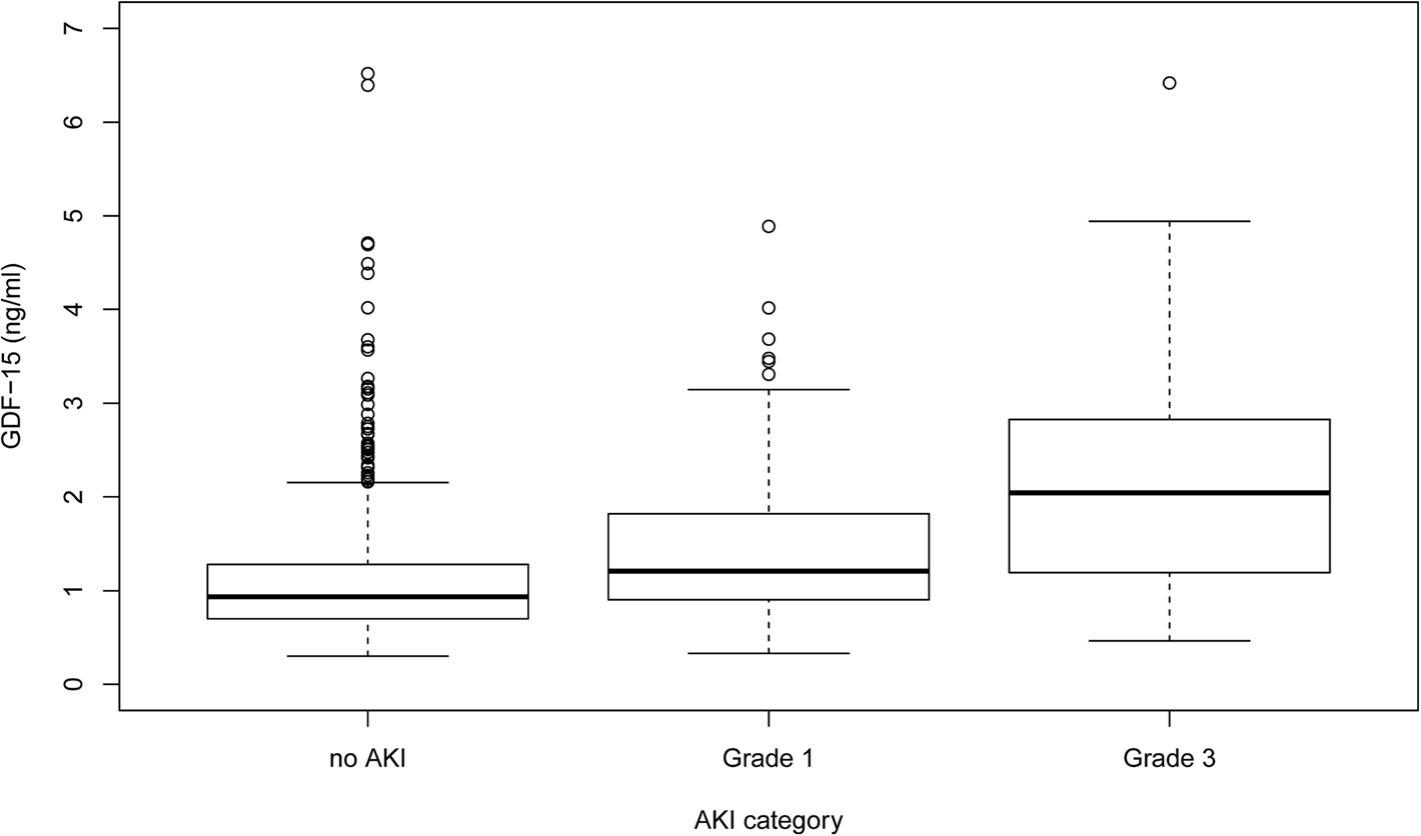
Plasma growth-differentiation factor-15 (*GDF-15*) in patients without acute kidney injury (*AKI*) and in patients with AKI grade 1 (AKI-1) and grade 3: *analysis of variance; *p* < 0.001). Preoperative plasma GDF-15 was significantly higher in relation to the severity of AKI in comparison with patients without this complication (*no AKI* vs. *Grade 1*, *p* < 0.001; *Grade 1* vs *Grade 3*, *p* < 0.001)

Postoperative AKI was observed in 258 patients (21.9 %) (AKI stage 1 (AKI-1), n = 175 (14.9 %); AKI-2, n = 6 (0.5 %); AKI-3, n = 77 (6.5 %)). The incidence of AKI-1 and AKI-3 increased significantly from the lowest to the highest tertiles of GDF-15 (Table 1). Accordingly, preoperative plasma GDF-15 was significantly higher in relation to the severity of AKI in comparison with patients without this complication (Fig. 1).

**Table 1:** Demographics, preoperative, operative and postoperative characteristics of the patient population according to tertiles of preoperative growth-differentiation factor-15

	GDF tertile 1	GDF tertile 2	GDF tertile 3	Total	*P* value
*N*	392	392	392	1176	
Male	282 (71.9 %)	272 (69.4 %)	256 (65.3 %)	810 (68.9 %)	0.129
Age (years)	59 (50/67)	69 (63/74)	72 (67/77)	68 (50/74)	<0.001
NYHA I	161 (41.1 %)	137 (34.9 %)	113 (28.8 %)	411 (34.9 %)	0.002
NYHA II	104 (26.5 %)	97 (24.7 %)	87 (22.2 %)	288 (24.5 %)	0.365
NYHA III	111 (28.3 %)	138 (35.2 %)	146 (37.2 %)	395 (33.6 %)	0.021
NYHA IV	15 (3.8 %)	19 (4.8 %)	44 (11.2 %)	78 (6.6 %)	<0.001
Additive Euroscore	3 (2/6)	5 (3/7)	7 (5/8)	5 (3/7)	<0.001
GDF-15 (ng/ml)	0.643 (0.535/0.730)	0.991 (0.914/1.114)	1.731 (1.438/2.329)	0.989 (0.729/1.435)	<0.001
NTproBNP (pg|ml)	187.8 (74.4/495.0)	427.9 (155.4/900.6)	1044.6 (383.4/2528.7)	434.5 (137.8/1139.3)	<0.001
hsTNT (pg/ml)	6.3 (3.0/11.1)	11.4 (6.6/18.8)	20.4 (12.0/38.0)	11.4 (5.6/21.5)	<0.001
ScO2_minox_ (%)	68 (63/72)	65 (60/70)	63 (57/67)	65 (60/70)	<0.001
Diabetes mellitus (*n* (%))	152 (38.8 %)	250 (63.8 %)	305 (77.8 %)	707 (60.1 %)	<0.001
LVEF 1 (*n* (%))	6 (1.5 %)	10 (2.6 %)	24 (6.1 %)	40 (3.4 %)	<0.001
LVEF 2 (*n* (%))	57 (14.5 %)	74 (18.9 %)	102 (26.0 %)	233 (19.8 %)	<0.001
LVEF 3 (*n* (%))	328 (36.4 %)	307 (34.1 %)	266 (29.5 %)	901 (76.8 %)	<0.001
Reoperation (*n* (%))	28 (7.1 %)	35 (8.9 %)	48 (12.2 %)	111 (9.4 %)	0.046
Creatinine (μmol/l)	73.9 (65.1/83.6)	82.7 (68.6/93.3)	93.3 (76.6/117.0)	81.0 (68.6 /96.8)	<0.001
eGFR (MDRD) (ml/min/m^2^)	94.2 (80.1/110.5)	80.7 (66.8/95.1)	66.2 (49.5/83.4)	80.9 (64.7/98.9))	<0.001
Hemoglobin (g/l)	139 (130/148)	135 (125/145)	129 (115/140)	135 (124/144)	<0.001
Peripheral vascular disease (*n* (%))	45 (11.5 %)	45 (11.5 %)	47 (12.0 %)	137 (11.6 %)	0.967
CPB time (minutes)	108 (86/144)	109 (84/135)	114 (92/149)	110 (88/142)	0.027
DHCA (*n* (%))	19 (4.8 %)	8 (2.0 %)	3 (0.8 %)	30 (2.6 %)	0.001
IOP hemofiltration (*n* (%))	12 (3.0 %)	12 (3.0 %)	36 (9.2 %)	60 (5.1 %)	<0.001
Isolated CABG (*n* (%))	173 (44.1 %)	199 (50.8 %)	164 (41.8 %)	536 (45.6 %)	0.033
Mitral valve surgery (*n* (%))	36 (9.2 %)	55 (14.0 %)	83 (21.2 %)	174 (14.8 %)	<0.001
Aortic valve surgery (*n* (%))	161 (41.1 %)	133 (33.9 %)	161 (41.1 %)	455 (38.7 %)	0.06
MAZE (*n* (%))	19 (4.8 %)	31 (7.9 %)	53 (13.5 %)	103	<0.001
HDU LOS (days)	2 (2/4)	3 (2/5)	4 (2/7)	3 (2/5)	<0.001
AKI 1	37 (9.4 %)	53 (13.5 %)	85 (21.7 %)	175 (14.9 %)	<0.001
AKI 2	3 (0.76 %)	1 (0.26 %)	2 (0.51 %)	6 (0.51 %)	0.65
AKI 3	4 (1.0 %)	17 (4.3 %)	56 (14.3 %)	77 (6.54 %)	<0.001
Renal replacement therapy	4 (1 %)	17 (4.3 %)	55 (14.3 %)	77 (6.45 %)	<0.001
30-day mortality	2 (0.5 %)	4 (1 %)	17 (4.4 %)	23 (1.96 %)	<0.001

*NYHA* New York Heart Association grade of heart failure, *NTproBNP* N-terminal pro-hormone of the B-type natriuretic peptide, *hsTN*T high-sensitivity troponine-T, *LVEF* left ventricular ejection fraction (1: <30 % or severely reduced; 2: 30–50 % or moderately reduced; 3: ≥50 % or normal), *MDRD* creatinine clearance according to the Modifications of Diet in Renal Disease formula, *CPB* cardiopulmonary bypass time, *DHCA* deep hypothermic circulatory arrest, *IOP-hemofiltration* intraoperative hemofiltration during CPB, *CABG* coronary artery bypass graft, *MAZE* MAZE - procedure; *HDU LOS* high-dependency unit time (intensive care and intermediate care unit), *AKI 1* to *AKI 3* acute kidney injury according to KDIGO creatinine criteria

Preoperative, operative, and postoperative characteristics are presented in Table 1, showing that patients in the highest GDF-15 tertile also had a significantly increased risk profile.

**Fig. 2:**
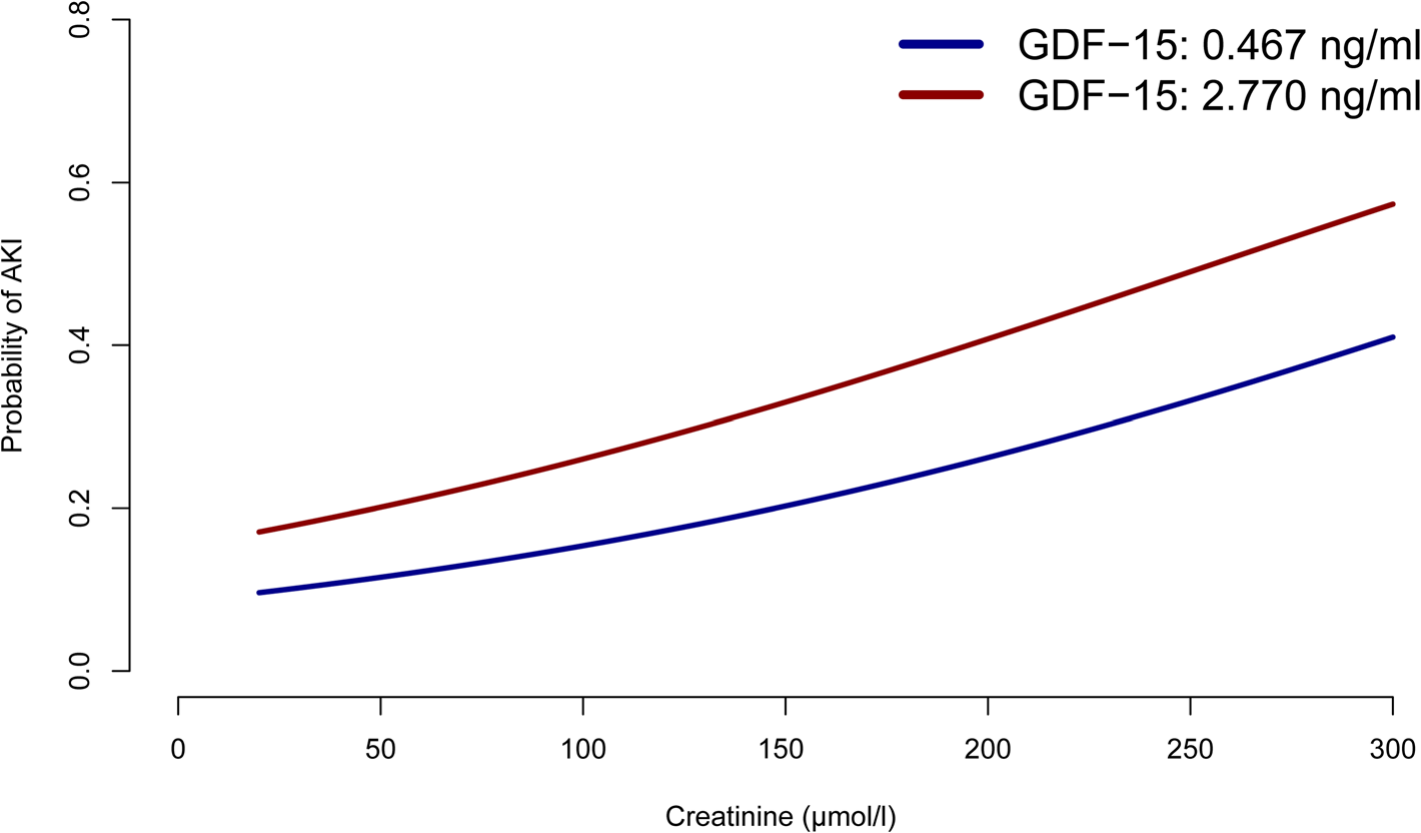
Probability of a prototypical patient aged 65 years with an additive Euroscore of 5.15, and a duration of cardiopulmonary bypass of 123 minutes for a range of creatinine values (*x-axis*) and the 5 and 95 % quantiles of growth-differentiation factor-15 (*GDF-15*) (0.467 and 2.770, respectively) as derived from the multivariate model. *AKI* acute kidney injury, *CC-ARF* Cleveland Clinic Acute Renal Failure (score)

The odds ratio from the final logistic regression model, which included GDF-15, was 1.314 (95 % CI 1.142, 1.551; *p* = 0.001), with bootstrap reliability of 95.4 %. Preoperative plasma GDF-15 was independently and significantly associated with the postoperative development of CSA-AKI. This was not the case for NTproBNP and hsTNT. The full model is presented in Table 2. To further elucidate the effects of adding GDF-15 to the multivariate model, the probability of AKI for a prototype patient is graphically displayed in Fig. 2.

**Table 2:** Final logistic regression model specification for any grade of acute kidney injury

Factor	Odds ratio	95 % CI	*P* value	Bootstrap reliability
Intercept	0.002	0.0006, 0.007	<0.001	99.8 %
GDF-15 (ng/ml)	1.314	1.142, 1.551	<0.001	95.4 %
Age (years)	1.039	1.021, 1.058	<0.001	98.9 %
Additive Euroscore	1.074	1.009, 1.143	0.02	71.3 %
Creatinine (μmol/l)	1.007	1.002, 1.011	0.003	79.0 %
Diabetes mellitus	1.362	0.981, 1.881	0.06	50.1 %
CPB time (minutes)	1.006	1.003, 1.009	<0.001	96.6 %

For any increase in growth-differentiation factor-15 (GDF-15) of 1 ng/ml the odds ratio for developing acute kidney injury of is 1.34. *CPB* cardiopulmonary bypass

**Fig. 3:**
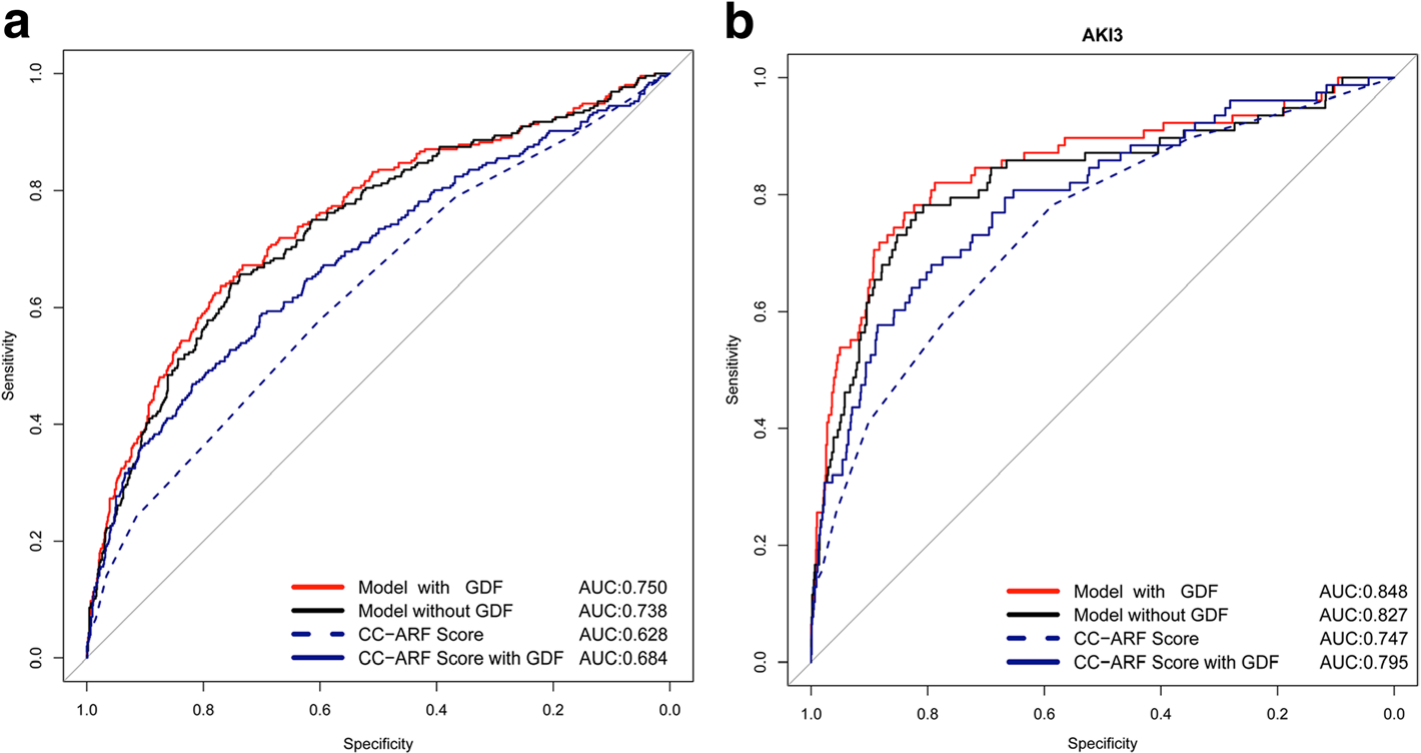
Receiver operator characteristics of the Cleveland clinic acute renal failure (*CC-ARF*) score [13] and nested logistic regression models on the development of cardiac-surgery-associated acute kidney injury (CSA-AKI) with and without taking into account preoperative plasma levels of growth-differentiation factor-15 (*GDF*). **a** Analyses for any stage of AKI (i.e., AKI-1 to AKI-3). **b** Analysis restricted AKI-3 only. The inclusion of GDF in the models led to statistically significant (analysis of variance; *p* < 0.001) predictive ability (either CC-ARF or a model based on additive Euroscore, age, plasma creatinine, diabetes mellitus, and duration of cardiopulmonary bypass). *AUC* area under the curve

In logistic regression analysis , GDF-15 was comparably and significantly associated with 30-day mortality (Additional file 1: Table S1). The ROC analysis of the CC-ARF score and the final logistic regression model - with and without the inclusion of GDF-15 - as a predictor variable is presented in Fig. 3a and b. In ROC analyses the CC-ARF score had only moderate predictive capacity (area under the curve (AUC) 0.628) for AKI-1 to AKI-3, which was increased by adding GDF-15 (AUC 0.684; *p* < 0.001). Our logistic regression model investigating the association between preoperative factors and all forms of postoperative AKI (AKI-1 to AKI-3) produced an AUC of 0.738, which was significantly increased (*p* < 0.001) to 0.750 by adding GDF-15 (*p* = 0.014).

**Fig. 4:**
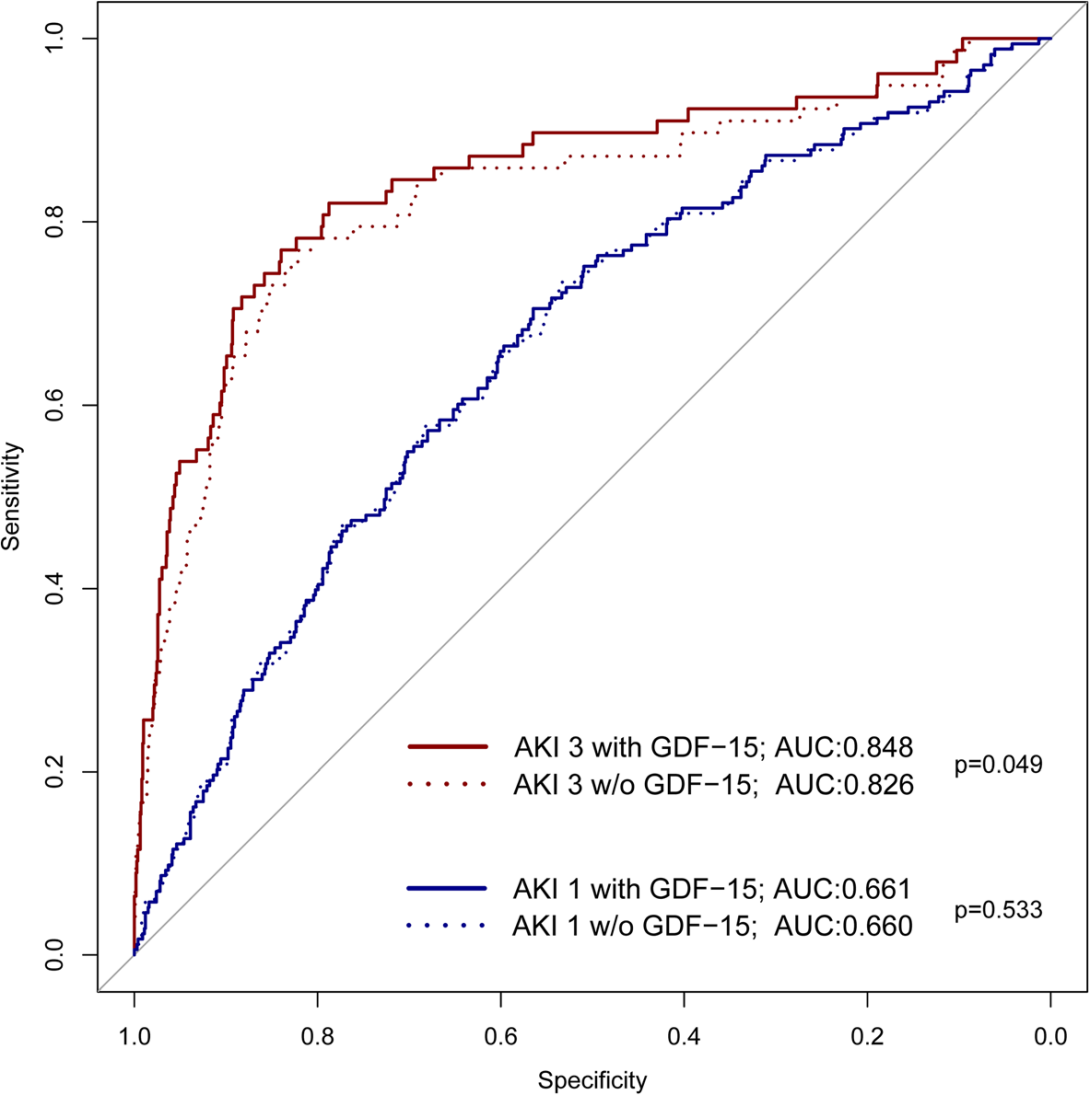
Receiver operator characteristics of nested logistic regression models on the development of cardiac-surgery-associated acute kidney injury (CSA-AKI) with and without (*w/o*) taking into account preoperative plasma levels of growth-differentiation factor-15 (*GDF*) for acute kidney injury (*AKI*) grade 1 and 3. While the inclusion of GDF led to a statistically significant increase in the area under curve (*AUC*) for AKI grade 3, this was not the case for AKI grade 1

On analysis restricted to the outcome postoperative AKI-3, the CC-ARF score alone had an AUC of 0.747, which was increased by GDF-15 to 0.795 (*p* < 0.001). In contrast the AUC of our clinical regression model for AKI 3 was 0.827, which was improved (*p* = 0.04) to an AUC of 0.848 by incorporating GDF-15. Such improvement in risk stratification was not observed in predicting AKI-1, as the addition of GDF-15 did not improve the AUC for AKI-1 (Fig. 4).

Reclassification analysis revealed that the addition of GDF-15 in the logistic regression model led to a statistically significant increase in the net reclassification ability. The reclassification analysis and tables are presented in Table 3.

**Table 3:** Reclassification analysis

Outcome: absent					
Updated model (risk categories)					
Initial model (risk categories)	(0, 0.01)	(0.01, 0.05)	(0.05, 0.1)	(0.1, 1)	% reclassified
(0, 0.01)	0	0	0	0	-
(0.01, 0.05)	0	53	2	0	4
(0.05, 0.1)	0	12	143	13	15
(0.1, 1) 0	0	24		669	3
Outcome: present					
Updated model (risk categories)					
Initial model (risk categories)	(0, 0.01)	(0.01, 0.05)	(0.05, 0.1)	(0.1, 1)	% reclassified
(0, 0.01)	0	0	0	0	-
(0.01, 0.05)	0	4	1	0	20
(0.05, 0.1)	0	1	16	1	11
(0.1, 1)	0	0	1	233	0
Combined data					
Updated model (risk categories)					
Initial model (risk categories)	(0, 0.01)	(0.01, 0.05)	(0.05, 0.1)	(0.1, 1)	% reclassified
(0, 0.01)	0	0	0	0	-
(0.01, 0.05)	0	57	3	0	5
(0.05, 0.1)	0	13	159	14	15
(0.1, 1)	0	0	25	902	3

Net-reclassification improvement (NRI) (categorical) (95 % CI): 0.0229 (0.0014, 0.0445); *p* value 0.03697. NRI (continuous) (95 % CI): 0.308 (0.1739, 0.4421); *p* value: 0.00001. Integrated discrimination improvement (95 % CI): 0.015 (0.006, 0.024); *p* value: 0.00107. Reclassification table of the model without (initial) and with (updated) growth-differentiation factor-15 (GDF-15) as a predictor of cardiac-surgery-associated acute kidney injury (CSA-AKI). The addition of GDF-15 in the logistic regression model significantly improved prediction of the development of CSA-AKI.

**Fig. 5:**
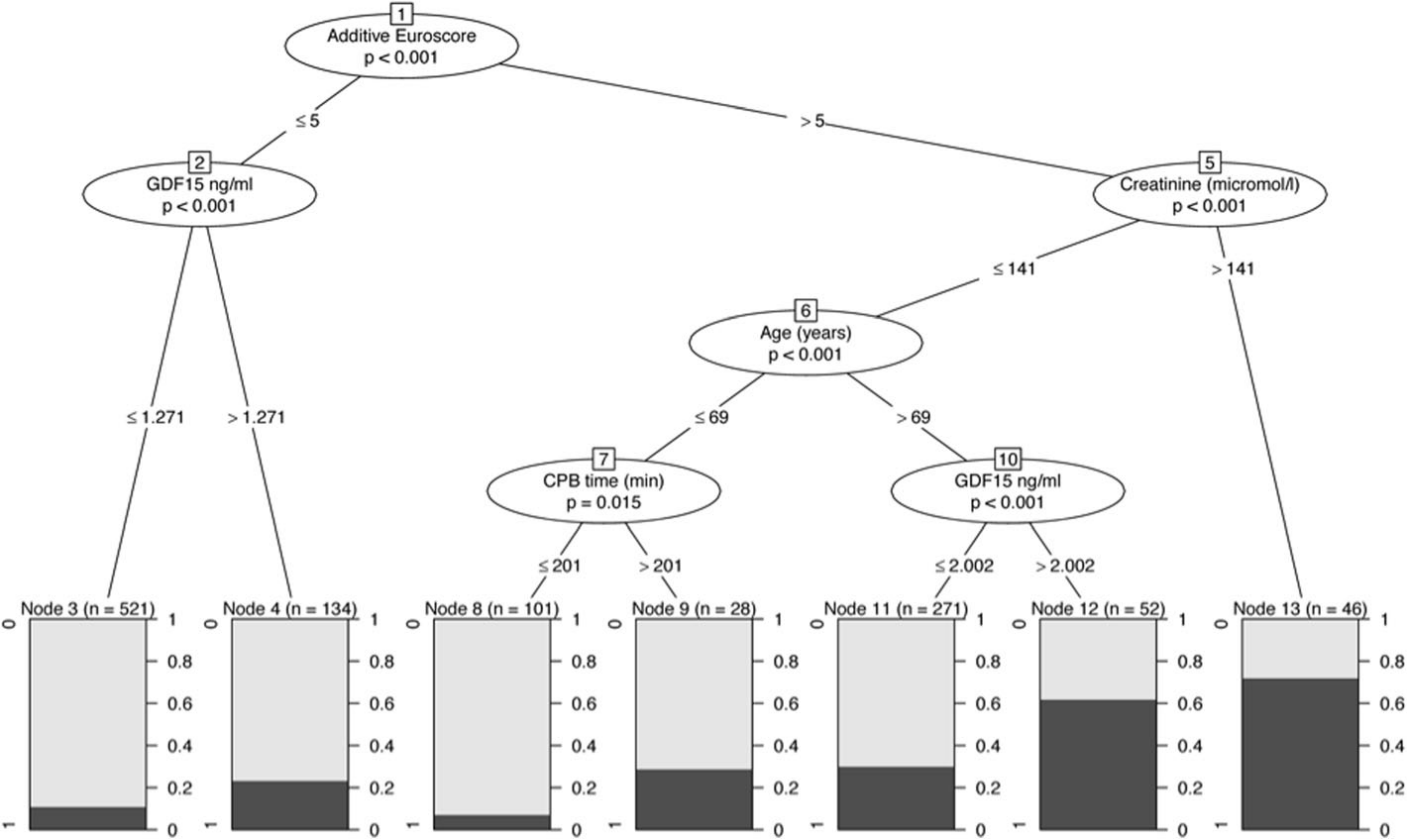
Recursive partitioning using conditional inference trees. Starting from the *top*, important variables and their respective cutoffs are presented leading (*bottom*) to the percentage of patients (within each population partition) developing cardiac-surgery-associated acute kidney injury (CSA-AKI). For example, from the group of patients with additive Euroscore <5 and growth-differentiation factor-15 (*GDF-15*) <1.271 ng/ml, 10 % developed CSA-AKI (*Node 3*), whereas from the group of patients with additive Euroscore <5 and GDF-15 > 1.271 ng/ml, 20 % of patients developed CSA-AKI (*Node 4*). *CPB* cardiopulmonary bypass

Random forest analysis showed that patient age, preoperative creatinine and preoperative plasma GDF-15 were the three most important variables associated with the development of CSA-AKI (Additional file 2: Figure S1). There was significant non-linearity and interactions of predictor variables in the incidence of AKI. As depicted in Additional file 3: Figure S2, within the same additive Euroscore category (panel row), increases in GDF (columns from left to right) are associated with increase in probability for CSA-AKI. As shown, this is especially pronounced in patients with creatinine levels <180 μmol/l. In addition, recursive partitioning revealed the important role of GDF-15 and specific plasma levels of this hormone stratified according to other independent risk factors (Fig. 5).

## Discussion

Several mechanisms mediating a perioperative decrease in renal function have been identified within recent years and several biomarkers have been proposed to facilitate early detection of AKI, i.e., neutrophil-gelatinase-associated lipocalin (NGAL), kidney-injury molecule -1 (KIM-1), liver-type fatty acid binding protein (L-FABP), interleukin-18 (IL-18), insulin-like growth factor-binding protein 7 (IGFBP7), and tissue inhibitor of metalloproteinase (TIMP-2) [17]. However, these biomarkers are intended for the early detection of AKI after a renal insult has occurred and not for preoperative risk stratification.

Extending the observations of two recent pilot studies in patients undergoing CABG [8, 9], the findings of the present study again show that preoperative plasma GDF-15 is an independent predictor of postoperative renal dysfunction in a heterogeneous population of patients undergoing elective cardiac surgery.

GDF-15, also entitled macrophage inhibitory cytokine-1 (MIC-1) is a cytokine expressed in many tissues, including myocardium, lung, kidney, brain, liver, and the intestine, upon various stimuli, including myocardial stretch, volume overload, experimental cardiomyopathy and oxidative stress, other inflammatory cytokines, and ischemia/reperfusion (for a detailed overview see [18]). However, the physiological role of this peptide in the cardiovascular system still remains to be defined.

Our group has recently shown that preoperative plasma GDF-15 is an independent predictor of postoperative mortality and morbidity in patients undergoing cardiac surgery and can further stratify patients beyond the established risk scores such as the Euroscore, and other cardiovascular risk markers such as NTproBNP or hsTNT [10]. The present analysis extends these findings to the prediction of CSA-AKI, an important complication in patients undergoing cardiac surgery, which is associated with poor short-term and long-term prognosis [1].

Employing logistic regression modeling of variables with an established (age, gender, additive Euroscore, serum creatinine, duration of CPB, duration of surgery, type of surgery, total circulatory arrest, preoperative hemoglobin, and diabetes mellitus) or putative (ScO_2_, hemofiltration during ECC, plasma GDF-15, hsTNT, and NTproBNP) role as risk factors for CSA-AKI, we observed that GDF-15 is an independent predictor of CSA-AKI and confirmed this finding using multiple statistical methods. It is of note that in random forest analysis the ability of GDF-15 to predict CSA-AKI was especially pronounced in patients with normal plasma creatinine; one explanation why this hormone had superior predictive ability in comparison with a conventional risk score like the additive Euroscore in our previous study [10]. Additionally, the observation that NTproBNP and hsTNT - despite being widely accepted biomarkers of cardiopulmonary dysfunction – did not predict AKI, further supports the powerful potential of GDF-15 for risk stratification in this regard. It is of note that the risk prediction potential of GDF-15 was primarily related to the ability to predict AKI-3. Whether this may be related to the physiology or pathophysiology of GDF-15 or that AKI-1 events are very difficult to predict remains speculative.

Various clinical scores for the prediction of renal dysfunction after cardiac surgery have been developed within recent years and these have highly variable predictive ability [7]. We tested the CC-ARF score as one of the most popular scores [13]. As expected, the predictive ability of this score, which was primarily developed to predict postoperative need of dialysis (that renders patients AKI stage 3), was rather poor if used to predict any type of AKI. However, when combined with GDF-15, the predictive ability was markedly improved for any kind of AKI and especially for AKI-3, as the most severe stage of postoperative renal dysfunction. This may have clinical relevance, because the CC-ARF score - in contrast to our model - has been externally validated and is widely used [13].

Very recently, Bignami and coworkers [19] reported that the preoperative plasma level of the endogeneous hormone ouabain is an independent predictor of AKI in a derivation and a validation cohort of patients undergoing cardiac surgery, and that it improves the predictive ability of a clinical risk score for AKI. It is of note that ouabain and GDF-15 both reflect circulatory stress [20, 21], supporting the role of this factor as a trigger of AKI in this setting. But there are also relevant discrepancies between the two peptides. First of all, Bignami et al. provided experimental data for a pathophysiological link between increased circulating levels of ouabain and decreased renal function (i.e., decreased creatinine clearance, increased urinary protein excretion, and reduced podocyte nephrin) [19], whereas a direct detrimental effect of increased GDF-15 levels on renal function has not been shown so far. In contrast, some lines of evidence point to a protective role of GDF-15 in diabetic nephropathy [22].

Additionally, Bignami et al. [19] employed a more rigorous definition of AKI (AKI grade 2 and 3) than we did. However, restricting our analyses to AKI-3 we also observed a numerically almost comparable and relevant increase in the AUC in ROC analysis by adding GDF-15 to our logistic regression model. Future studies need to determine which of these peptides has the better power to predict for all stages and the most severe forms of AKI.

## Limitations

The present study has several limitations. First, this is a secondary analysis of a monocentrical, observational study primarily aiming to determine the association between GDF-15 and postoperative morbidity and mortality. Consequently, as we have shown that there is such an association (i.e., that preoperative GDF-15 is an independent marker of morbidity and mortality in this cohort of patients [10]) it cannot be ruled out completely that the described association between preoperative GDF-15 and AKI is epiphenomenal. This may also be true for the observed association of a higher clinical risk profile and GDF-15 tertiles and the observation, that - also in this cohort of patients undergoing elective surgery - GDF-15 was an independent predictor of 30-day mortality. With respect to the high mortality in AKI [1], cross-correlation between these two outcomes is almost inevitable. However, the results of the random forest analysis - showing that GDF-15 is especially useful for predicting AKI in patients with low plasma creatinine and who do not typically have a high risk profile - indicates that there are subgroups of patients in whom such an epiphenomenal association is at least not obvious.

As a second point one may argue that the improvements in the clinical models by incorporating GDF-15 were numerically small and despite being statistically significant, they may be of questionable clinical relevance. Nonetheless, the reclassification analyses clearly show that the net effect of reclassification taking into account GDF-15 levels in comparison with the logistic regression model alone was much more pronounced than suggested by the small differences in the AUC [16].

Third, the CC-ARF score had significantly lower ability to predict AKI-1 to AKI-3 and AKI-3 alone than logistic regression analysis based on the present cohort. This contrasts with some studies showing excellent prediction of AKI using the CC-ARF score [7, 13] This, however may at least in part be explained by the fact that the present cohort consisted only of patients undergoing elective surgery, as emergency patients were excluded. Thus, the difference between our model and the CC-ARF score may be less pronounced during real-life conditions.

Additionally, we classified AKI only according to creatinine criteria, because data on urine flow were only available for patients studied in 2009. This may lead to discrepancies in comparison with studies analyzing the predictability of renal risk scores based on the analysis of creatinine and urine flow. Recent data from critically ill patients [23] and patients undergoing cardiac surgery [24] clearly suggest that the omission of urine flow may have led to underestimation of the incidence of AKI. However, patients diagnosed with AKI according to creatinine criteria seem to have a much worse prognosis, i.e., higher mortality [23, 24]. Consequently, AKI diagnosis based only on creatinine criteria may be regarded as more conservative and helpful in identifying those patients with renal dysfunction who have the highest mortality risk.

Fourth, despite confirmation of findings from smaller pilot studies by the present analysis, definitive confirmation of the role of GDF-15 for predicting AKI mandates further and multicenter prospective trials. Ideally, these studies should specifically address diabetes mellitus as a potential confounder, because patients with diabetes mellitus also have increased GDF-15 [22] and high risk of AKI after cardiac surgery [1, 5]. It is of note that the number of patients with diabetes mellitus in the present study in the highest GDF-15 tertile was almost twice as high than in the lowest GDF-15 tertile.

As a last and more general point, it has to be taken into account that a preoperative score or biomarker will never be able to perfectly predict a multifactorial complication like CSA-AKI, because unpredictable intraoperative and postoperative factors, like unexpected prolongation of surgery or prolonged mechanical ventilation [6], may render any prediction model - at least partially - imprecise.

## Conclusions

In conclusion and taking into account the limitations of a monocentric study, but supporting findings from previous work in patients undergoing CABG [8, 9], the present analysis shows that preoperative plasma GDF-15 is an independent predictor of postoperative AKI in patients undergoing elective cardiac surgery, and improves the predictive ability of the CC-ARF score as an established renal risk score and of logistic regression models based on the additive Euroscore, age, duration of CPB, and diabetes mellitus. Additionally, this biomarker seems to be particularly helpful for further risk stratification beyond accepted risk factors, i.e., especially in patients with low preoperative creatinine.

## Key messages

- Acute kidney injury (AKI) is a serious and frequent complication in patients undergoing cardiac surgery
- Growth-differentiation factor-15 (GDF-15) is a cytokine expressed upon myocardial stretch and volume overload, and during oxidative stress and ischemia/reperfusion
- GDF-15 has been shown to be reflective of poor prognosis in various clinical settings, including heart failure, myocardial infarction, and in patients undergoing cardiac surgery
- The present study shows that preoperative plasma GDF-15 is an independent predictor of postoperative AKI in patients undergoing elective cardiac surgery and improves the predictive ability of the established renal risk score, the Cleveland Clinic Acute Renal Failure score and of an individual logistic regression model based on the additive Euroscore, age, duration of CPB, and diabetes mellitus

## Additional files

Additional file 1: Logistic regression model specification for 30 day mortality. (DOCX 27 kb)
Additional file 2: Figure S1. Visualization of the predictive ability of each input variable on the development of Cardiac Surgery-associated Acute Kidney injury (CSA-AKI) (random forest model). Variables with greater predictive ability (growth-differentiation factor-15 (GDF-15), age) exhibit high variable importance and low minimal variable depth, whereas variables less associated with CSA-AKI (diabetes) exhibit low variable importance and high minimal depth. (TIFF 2787 kb)
Additional file 3: Figure S2. Covariate plot of the predictions from the random forest model. Four variables are displayed. Within each *box*, the *x-axis* denotes plasma creatinine (μmol/l), the *y-axis* the probability of developing cardiac-surgery-associated acute kidney injury (CSA-AKI). Within each *column* of plots, the additive Euroscore increases from *bottom* to *top* (*legend*, *right*). Within each *row* of plots the growth-differentiation factor-15 (GDF-15) levels increase from *left* to *right*. Significant non-linear interaction takes place between GDF-15 and creatinine. Within each additive Euroscore category (*row* of plots), increases in GDF-15 (plots more to the *right* within each row) increase the probability of developing CSA-AKI. However, this effect is more prominent in patients with normal creatinine (*x-axis label* of each plot). (TIFF 3197 kb)

## Abbreviations

AKI: Acute kidney injury
AUC: Area under the curve
CABG: Coronary artery bypass graft
CC-ARF: Cleveland Clinic Acute Renal Failure (score)
CPB: Cardiopulmonary bypass
CSA-AKI: Cardiac surgery associated acute kidney injury
eGFR: Estimated glomerular filtration rate (creatinine clearance)
GDF-15: Growth-differentiation factor-15
hsTNT: High sensitivity Troponin T
KDIGO: Kidney Disease Improving Global Outcomes
MIC-1: Macrophage inhibitory cytokine-1
NTproBNP: N-terminal prohormone of the B-type natriuretic peptide
ROC: Receiver-operating characteristic
ScO_2_: Cerebral oxygen saturation
