# Supplementary material for: Preoperative plasma growth-differentiation factor-15 for prediction of acute kidney injury in patients undergoing cardiac surgery
Source: Crit Care. 2016 Oct 8;20:317. doi: 10.1186/s13054-016-1482-3 (PMC5055664; doi:10.1186/s13054-016-1482-3)
Supplement: Additional file 1: — Logistic regression model specification for 30 day mortality. (DOCX 27 kb) [file 13054_2016_1482_MOESM1_ESM.docx]

Table S1

| Variable | Estimate | 95% CI | P value |
| --- | --- | --- | --- |
| Intercept | 0.000001 | 0-0.001 | <0.0001 |
| GDF15 (ng/ml) | 1.486 | 1.270-1.792 | <0.0001 |
| Age (years) | 1.110 | 1.04 – 1.19 | 0.003 |
| CPB time | 1.007 | 1 – 1.01 | 0.05 |

The logistic regression model specification for 30 day mortality. GDF-15: Growth differentiation factor-15; CPB: cardiopulmonary bypass. For any increase in GDF-15 of 1 ng/ml there is an Odds ratio of 1.486 not to survive for more than 30 days.
