# Supplementary figures and images for: Preoperative plasma growth-differentiation factor-15 for prediction of acute kidney injury in patients undergoing cardiac surgery
Source: Crit Care. 2016 Oct 8;20:317. doi: 10.1186/s13054-016-1482-3 (PMC5055664; doi:10.1186/s13054-016-1482-3)

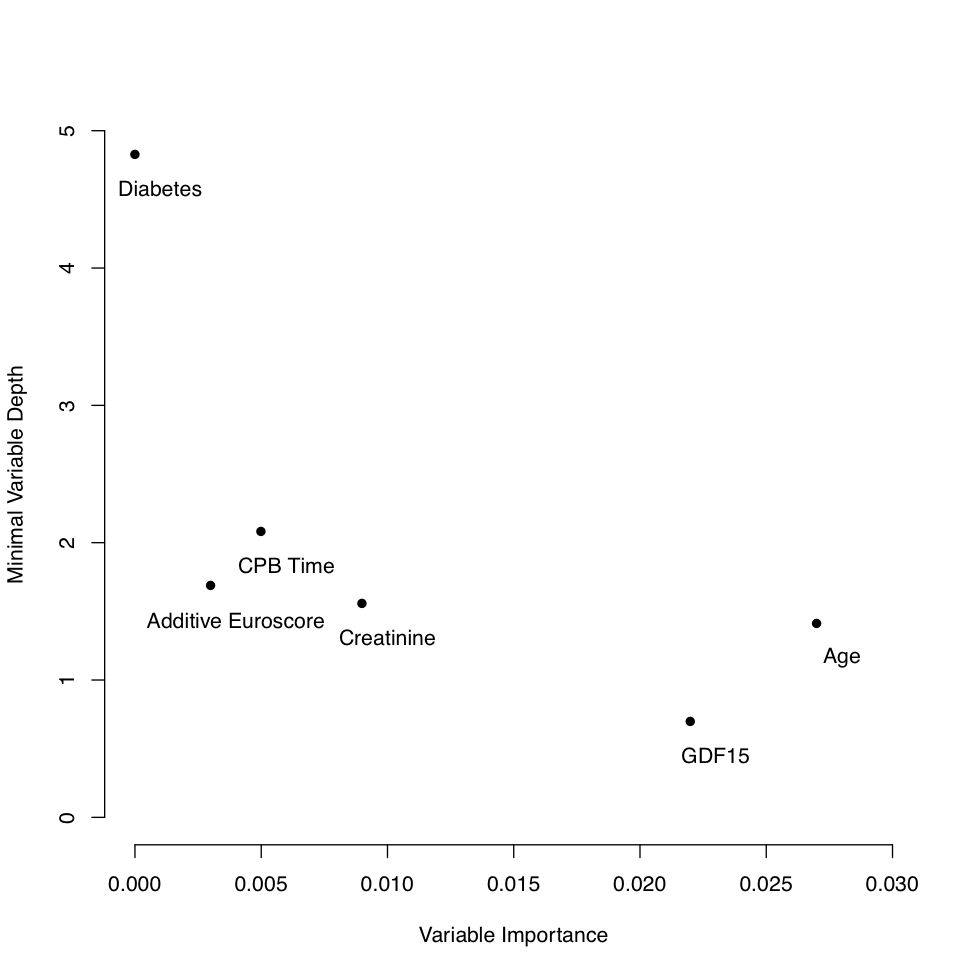

Supplement: Additional file 2: Figure S1. — Visualization of the predictive ability of each input variable on the development of Cardiac Surgery-associated Acute Kidney injury (CSA-AKI) (random forest model). Variables with greater predictive ability (growth-differentiation factor-15 (GDF-15), age) exhibit high variable importance and low minimal variable depth, whereas variables less associated with CSA-AKI (diabetes) exhibit low variable importance and high minimal depth. (TIFF 2787 kb) [file 13054_2016_1482_MOESM2_ESM.tiff]

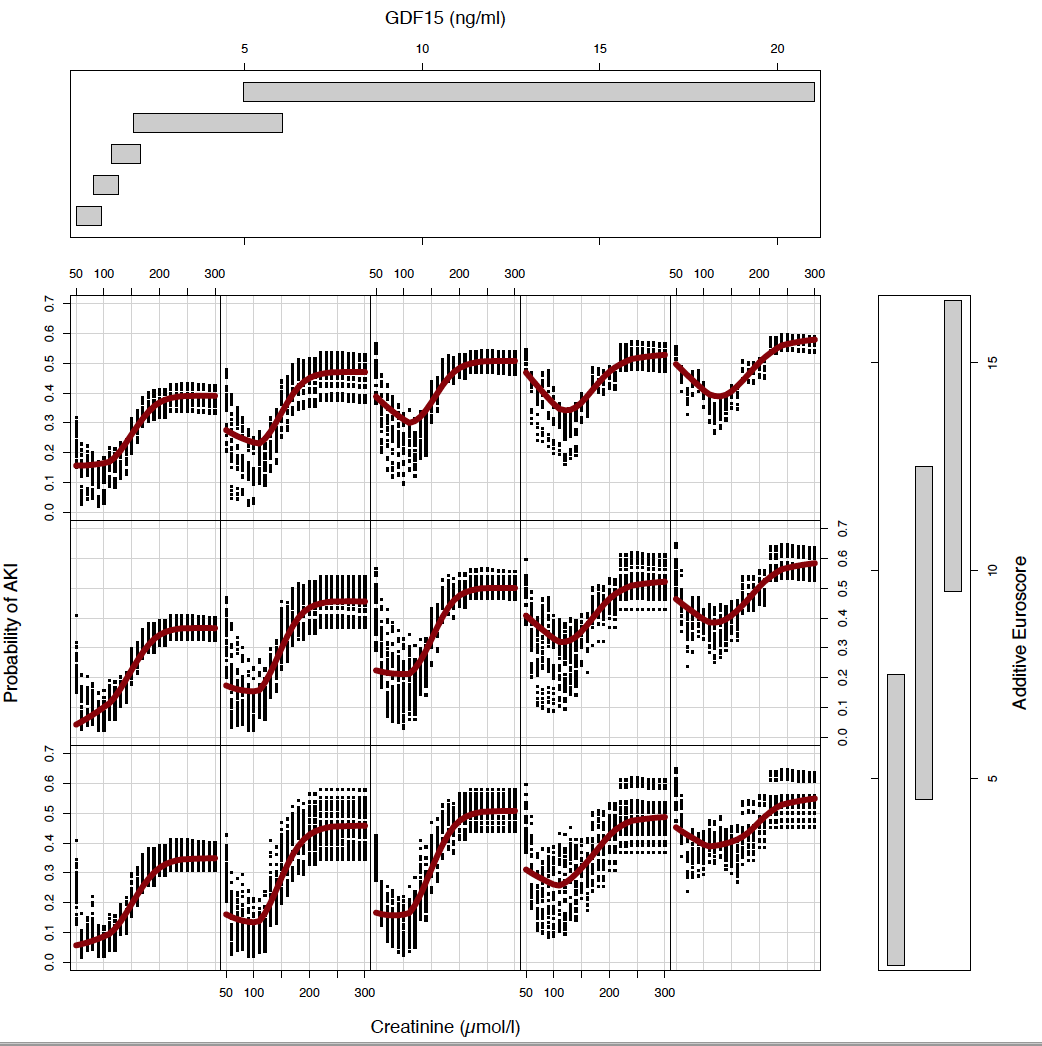

Supplement: Additional file 3: Figure S2. — Covariate plot of the predictions from the random forest model. Four variables are displayed. Within each box, the x-axis denotes plasma creatinine (μmol/l), the y-axis the probability of developing cardiac-surgery-associated acute kidney injury (CSA-AKI). Within each column of plots, the additive Euroscore increases from bottom to top (legend, right). Within each row of plots the growth-differentiation factor-15 (GDF-15) levels increase from left to right. Significant non-linear interaction takes place between GDF-15 and creatinine. Within each additive Euroscore category (row of plots), increases in GDF-15 (plots more to the right within each row) increase the probability of developing CSA-AKI. However, this effect is more prominent in patients with normal creatinine (x-axis label of each plot). (TIFF 3197 kb) [file 13054_2016_1482_MOESM3_ESM.tiff]
